# Plasticity-Inducing TMS Protocols to Investigate Somatosensory Control of Hand Function

**DOI:** 10.1155/2012/350574

**Published:** 2012-05-16

**Authors:** M. Jacobs, A. Premji, A. J. Nelson

**Affiliations:** ^1^Department of Kinesiology, University of Waterloo, 200 University Avenue West, Waterloo, ON, Canada N2L 3G1; ^2^Department of Medicine, University of Toronto, Toronto, ON, Canada M5S 1A1

## Abstract

Hand function depends on sensory feedback to direct an appropriate motor response. There is clear evidence that somatosensory cortices modulate motor behaviour and physiology within primary motor cortex. However, this information is mainly from research in animals and the bridge to human hand control is needed. Emerging evidence in humans supports the notion that somatosensory cortices modulate motor behaviour, physiology and sensory perception. Transcranial magnetic stimulation (TMS) allows for the investigation of primary and higher-order somatosensory cortices and their role in control of hand movement in humans. This review provides a summary of several TMS protocols in the investigation of hand control via the somatosensory cortices. TMS plasticity inducing protocols reviewed include paired associative stimulation, repetitive TMS, theta-burst stimulation as well as other techniques that aim to modulate cortical excitability in sensorimotor cortices. Although the discussed techniques may modulate cortical excitability, careful consideration of experimental design is needed to isolate factors that may interfere with desired results of the plasticity-inducing protocol, specifically events that may lead to metaplasticity within the targeted cortex.

## 1. Introduction

There is emerging evidence that alterations in somatosensory processing may underlie challenges in hand control after neurological injury. Abnormalities in somatosensory physiology and perception are observed in clinical populations such as stroke and focal hand dystonia [1, 2]. Evidence suggests that somatosensory-based therapies provide temporary benefits [3–5]. To translate fundamental science into effective therapies aimed at long-term improvements in hand function, a comprehensive understanding of the role of somatosensory cortex must be incorporated into models of hand control. In recent years, the use of plasticity-inducing transcranial magnetic stimulation (TMS) protocols has become a powerful tool to investigate the neural activity within the hand representations in primary somatosensory (SI) and primary motor (M1) cortices, touch perception and motor behavior. Such protocols have furthered our understanding of the somatosensory contributions to hand function.

Somatosensory input is represented in multiple cortical areas, similar to other sensory areas. The significance of multiple cortical representations of the hand remains unclear, although one hypothesis is that each area contributes a particular attribute to the process of sensory-guided movement [6]. Emphasis has largely been directed to understanding processing in SI. This information has served clinical neuroscience well as decades of monkey research have exposed fundamental principles of neural plasticity that have instructed formulas for rehabilitation training in patient groups [4]. SI which encompasses the postcentral gyrus is composed of at least four subareas in monkeys [7, 8] and humans [9, 10] that include 3a, 3b, 1, and 2. With the exception of 3b, evidence in monkey species demonstrates that all sub-areas project directly to M1 [11–18]. Higher-order somatic loci such as the secondary somatosensory cortex or Brodmann's area 5 share particular features such as large neural receptive fields and gross somatotopy [19, 20]. Area 5, located in the superior parietal lobule, is particularly interesting since it appears to be dominated by the representation of the hand and upper limb [21, 22] and is largely absent in species lacking opposable thumbs [21, 22] suggesting its emergence with skilled thumb manipulation [21]. The projection from area 5 to M1 is considered to be as dense as that which originates from SI [23].

There is substantial evidence that alterations in SI activity can affect somatosensation, M1 physiology, and motor control. In monkeys, manipulation of peripheral input through nerve crush or digit amputation [24] or experience [25] leads to neuroplastic changes within the postcentral gyrus. Lesions directly to SI such as the removal of the hand and arm representations within areas 1, 2, and 3 elevate detection thresholds by 3–6 times [26], while damage to area 1 leads to deficits in texture perception, and damage to area 2 impairs the percepts of curvature and form [27]. In monkeys, direct manipulation of SI activity alters motor behaviour. Injection of muscimol in the SI region leads to loss of finger coordination [28], cooling the postcentral gyrus leads to clumsy, slow movements, and poor coordination [29], and lesioning impairs the acquisition of new motor skills [30]. Direct manipulation of SI activity also alters neural responsiveness within M1. Tetanic stimulation applied to SI in cats increases the responsiveness in M1 neurons [31, 32]. Cooling the postcentral gyrus increases the background activity in M1 neurons, suggesting that SI may have a net inhibitory influence on M1 [29]. In another study, the SI versus M1 effects on EMG activity were compared using intracortical microstimulation during wrist movements. Compared to M1, stimulation to SI yielded a smaller percentage of neurons that altered EMG activity, and this change was more often suppression rather than facilitation of EMG activity [33]. Collectively, these data indicate that alterations to SI influence neural mechanisms that underpin somatosensory and motor processing.

In humans, understanding somatosensory physiology and its influence on M1 activity and motor control of the hand is important to basic and clinical neuroscience. This review is focussed on the use of plasticity-inducing TMS protocols to further the understanding of somatosensory contributions to hand function. Identifying methods to increase or decrease neural activity within hand representations may advance the development of therapies intended to improve hand function in clinical populations. We focus primarily on evidence that three TMS protocols applied to SI modulate perception and neural activity within SI and M1 and include paired associative stimulation, repetitive TMS and theta-burst TMS. For each, we briefly describe the neural mechanisms that appear to underpin effects, the influence on somatosensory and motor physiology, tactile perception and motor behaviour when delivered over SI, and the potential limitations of the technique. We subsequently describe additional paradigms that have also provided evidence of modulating SI activity.

## 2. TMS Plasticity-Inducing Protocols

Investigations into the somatosensory influence on M1 activity in humans often employ TMS paradigms to examine corticospinal excitability and specific neural circuitry. We briefly review specific TMS paradigms to measure such circuitry and refer the reader to other reviews intended to cover these topics more thoroughly [34, 35].

### 2.1. TMS Paradigms to Measure Corticospinal Excitability and Intracortical Circuitry

Single-pulse TMS delivered over a particular muscle representation within M1 at intensities above threshold can evoke a motor response in that contralateral muscle. The amplitude of the resultant motor-evoked potential (MEP) recorded from the target muscle reflects the excitability of corticospinal circuitry and spinal motoneurons [36–38]. Using dual-pulse TMS paradigms, intracortical inhibitory circuits within M1 may be tested by controlling the interstimulus interval (ISI) between the two TMS pulses delivered to M1. Short-interval intracortical inhibition (SICI) occurs when the ISI is in the range of 1–6 ms [39–41], and long-interval intracortical inhibition (LICI) occurs when the ISI is in the range of 50–200 ms [42]. In SICI and LICI, the first TMS pulse acts to inhibit the corticospinal output produced by the second TMS pulse such that the MEP amplitude is reduced. In contrast, an ISI in the range of 8–30 ms results in an increase in MEP amplitude, circuitry referred to as intracortical facilitation (ICF) [39]. ICF occurs when the first TMS pulse facilitates the corticospinal output produced by the second pulse [39, 41]. In addition to circuitry within M1, interhemispheric interactions between M1 in opposite hemispheres can be studied. Using two TMS coils, one TMS pulse called the conditioning stimulus (CS) is applied to M1 in one hemisphere and is followed by a TMS pulse called the test stimulus (TS) delivered to the opposite M1 [43]. This circuitry is known as interhemispheric inhibition (IHI) and is subdivided into short and long intervals with maximal inhibition at ~10 and 40 ms, respectively [44, 45]. Short- (SIHI-) and long- (LIHI-) interval IHI appear to mediated by different mechanisms since baclofen, a GABA_B_ receptor agonist, alters LIHI without changing SIHI [46]. In addition to circuitry probed within and between motor cortices, other neural interactions can be assessed by pairing peripheral nerve stimulation or cutaneous stimulation of the hand with a TMS pulse over M1. ISIs of approximately 20–50 ms or 200–1000 ms decrease motor excitability, effects known as short-latency afferent inhibition (SAI) or long-latency afferent inhibition (LAI), respectively [47–49].

#### 2.1.1. Paired Associative Stimulation

Paired associative stimulation (PAS) involves peripheral nerve stimulation followed by a TMS pulse delivered over the cortex, typically M1. Pairs of nerve-cortex stimuli are applied repetitively and result in long-lasting changes in cortical excitability [50]. Depending on the interstimulus interval (ISI) between the peripheral nerve stimulation and the TMS pulse, PAS can elicit changes in corticospinal excitability, indexed by increases or decreases in MEP amplitude, respectively. PAS applied with an ISI of 25 ms (PAS_25_) increases MEP amplitude [50, 51], while a 10 ms ISI (PAS_10_) decreases MEP amplitude [52]. Changes in cortical excitability persist for approximately 30 to 60 minutes following PAS [50, 53].

The PAS protocol was developed based on animal models of spike timing-dependent plasticity, particularly long-term potentiation (LTP)/depression (LTD) (for review, see [54, 55]). It has been proposed that PAS induces long-lasting changes in M1 circuitry via LTP/LTD-like mechanisms of cortical synapses, with the direction of excitability dependent on the interval between stimuli [50–52]. The bidirectional effects of PAS are dependent upon the temporal order of the paired stimuli. This timing is known to modulate the levels of postsynaptic calcium concentrations via N-methyl-D-aspartate (NMDA) receptor activation which ultimately determines the LTP- or LTD-like effects [54]. The arrival of peripheral afferent stimuli to the motor cortex via horizontal corticocortical fibres with the near simultaneous TMS pulse delivered over M1 leads to MEP facilitation suggesting LTP-like effects. In contrast, if the temporal order is reversed with the TMS pulse reaching the motor area first, the MEPs show LTD-like suppression. Temporal order is a feature associated with LTP/LTD [54]. Further, PAS effects localized to the target APB muscle do not carry over to muscle representations distant from the paired electrical inputs [56]. This is in line with LTP/LTD mechanisms thought to be associated with input specificity demonstrated in the rat motor cortex [54, 57]. Last, blocking NMDA receptor activity abolishes the PAS effects in humans [58, 59] and rats [60].

Long-lasting changes in M1 excitability are induced for up to an hour following the use of the PAS technique which combines somatosensory afferent input and direct modulation of cortical activity using TMS. Though several studies have demonstrated the influence of PAS on corticospinal excitability as measured via MEPs [50, 52], it appears that intracortical circuits are unaffected [51, 61]. However, using a lower-intensity PAS protocol whereby median nerve stimulation is paired with single-pulse TMS over M1 (i.e., intensity to elicit a 0.5 mV MEP versus 1 mV used in other studies), long-lasting changes in inhibitory intracortical circuits of M1 were observed [62]. Specifically, following PAS_25_, long-interval intracortical inhibition (LICI) was reduced and long-latency afferent inhibition (LAI) was reduced when the ISI between the CS and TS was 150 ms. In contrast, PAS_10_ increased LICI, while LAI showed a nonsignificant increase. Interestingly, at an ISI of 240, the direction of LAI reversed with PAS_25_ and PAS_10_, emphasizing the importance of intervals between stimulation. PAS_10_ decreased SICI, while the effects of PAS_25_ were inconsistent [62]. Overall, this work demonstrates that PAS, a technique that manipulates the arrival of the somatosensory afferent volley and M1 cortical activity, can indeed modulate intracortical circuitry within M1.

PAS has also been used to demonstrate changes in SI excitability. PAS may be performed by pairing median nerve stimulation with TMS pulses delivered directly over SI. The interval between the nerve and SI stimulation may be determined by using the individual latencies of the N20 SEP potential. Using this technique, intervals that aim to closely time the arrival of the afferent volley with the TMS pulse applied to SI lead to a facilitation of the P25 SEP. In contrast, when the SI TMS pulse is delivered in advance of the arrival of the afferent volley by ~20 ms, the P25 is decreased [63] in line with an inhibitory versus excitatory effect of short versus long intervals, respectively. However, a recent study was unable to replicate these findings [64] possibly related to the lower intensities used for the SI TMS pulse (120% versus 150% RMT) and median nerve stimulation (110% of motor threshold versus 300% perceptual threshold). Thus, it may be that PAS over SI may require higher TMS intensities than that required by PAS over M1. In one study using a similar paradigm, the effects of PAS on single- and paired-median nerve SEPs were evaluated in controls and patients with focal hand dystonia [65]. In contrast to the previous report, PAS did not significantly alter SEPs in the healthy control group. PAS did however increase SEPs and intracortical inhibition in FHD [66].

### 2.2. PAS Considerations

The facilitatory and inhibitory effects of PAS 25 and 10 ms are altered by prior neural activity. This effect known as metaplasticity describes a change in the neuroplasticity effects as a result of the recent history [67]. Preconditioning with 250 suprathreshold TMS pulses delivered at 0.1 Hz eliminates the PAS 25 facilitation and PAS 10 suppression. Subthreshold TMS and also median nerve stimulation preconditioning did not abolish the PAS- 25-induced facilitation though they did prevent the increase from achieving statistical significance [68]. Similarly, motor training involving the thumb blocks the PAS- 25-induced facilitation [69, 70] and either leaves PAS-induced inhibition unchanged [69] or enhances the inhibition [70]. These data suggest that limiting neural activity is an important determinant for observing PAS facilitation and inhibition, and that a prior history of activity within the neural targets of PAS will affect the outcome of this plasticity-inducing protocol.

The after effects of PAS seem to be more effective depending on the time of day, suggesting that circadian rhythms and hormonal fluctuations may influence the magnitude of PAS effects [71]. PAS was significantly more effective in the afternoon compared to morning sessions [71]. Having the subject directed their attention to the stimulated hand also increases the magnitude of PAS effects [72].

There is conflicting evidence of the effects of PAS on spinal circuit excitability. While authors report no changes in spinal excitability with the use of F-waves [56, 59], others have recently found changes at the spinal level with the use of H-reflex recruitment curves [73, 74]. The authors conclude that the PAS-induced increase of the H-reflex is due to a decrease in the presynaptic inhibition of Ia terminals [74]. It is important to note, however, that the latter group used a modified version of PAS in which the stimulation protocol was delivered at a faster rate of 0.2 Hz and applied 240 paired pulses [73, 74]. Facilitatory PAS protocols which deliver more stimuli at a higher rate [71] comparably induce greater increases in MEP amplitude than standard PAS [56]. It is possible that paired stimulus parameters of greater intensity, frequency, and number of pulses may induce a greater degree of descending modulation of spinal circuitry than standard PAS protocols.

#### 2.2.1. Repetitive TMS

TMS, applied repetitively, can be used to induce short-term changes in cortical excitability. The effects of repetitive TMS (rTMS) are dependent on the stimulus parameters of the protocol, with the main determinants being the frequency of pulse delivery and the intensity [75]. RTMS delivered at frequencies ≤1 Hz lead to the suppression of MEP amplitude [76] and increased MEP amplitude when applied at frequencies ≥1 Hz [77, 78]. RTMS has also been shown to alter intracortical circuitry such that SICI decreases [79].

The physiological basis of increases and decreases in cortical excitability with high- versus low-frequency rTMS has been attributed to LTP and LTD of cortical synapses [34, 80]. In rat models, 1 Hz rTMS reduced the number of calbindin D-28k- (CB-) positive cells suggesting that inhibitory activity of interneurons controlling synaptic input to pyramidal cells was altered [81]. Within two hours following 1 Hz rTMS, GAD 67 expression related to GABA synthesis in the cytosol was reduced, while GAD 65 related to GABA synthesis for neurotransmission was unaltered [82]. However, one to seven days following stimulation, GAD 65, GAD 67, and GAT1 GABA transport expression increased suggesting longer-term changes in inhibitory neural circuits [82].

In humans, Satow et al. (2003) investigated the influence of low-frequency rTMS (0.9 Hz) over the left-hemisphere motor hotspot for APB and at two alternate positions 3 cm anterior and 3 cm posterior to the hotspot. Effects were only induced at the APB site which the authors refer to as the “sensorimotor” site [83]. Somatosensory-evoked potentials (SEPs) were unchanged though thresholds for the detection of tactile stimuli using Von Frey filaments increased for 0–8 minutes on the right index finger [83]. Tactile frequency discrimination on the left hand was impaired following 1 Hz rTMS over right SI with the duration of impairment related to the duration of rTMS [84]. The longest perceptual impairment persisted for 8 minutes and occurred following twenty minutes of rTMS low-frequency rTMS [84]. Spatial acuity measured using 2-point discrimination is also impaired following 1 Hz rTMS over SI, although the effects on wrist proprioception were variable [85].

High-frequency rTMS applied to SI alters tactile acuity and physiology. Gains in tactile spatial acuity achieved using tactile coactivation paradigms were further improved by combining it with high-frequency rTMS (5 Hz) over SI [86]. A subsequent report by this group used paired-median nerve stimulation whereby 5 Hz rTMS over SI reduced the inhibition of the second SEP within the pair [87]. 5 Hz rTMS applied over SI was subsequently shown to improve tactile spatial acuity on the index finger and increase the representation of that finger within SI [88]. Similar findings for tactile frequency discrimination and enlarged cortical activation within SI were subsequently reported [89]. There is also evidence to suggest that high-frequency rTMS facilitates somatosensory learning. The learning of a spatial discrimination task was improved when paired with high-frequency (15 Hz) rTMS over SI. In contrast, rTMS did not improve the learning of a frequency discrimination task [90].

In humans, rTMS has been applied to SI to investigate the after effects on motor behaviour and M1 physiology. Vidoni et al. (2010) studied the influence of low-frequency rTMS over SI on the ability to learn a motor tracking task. Real and sham 1 Hz rTMS was applied over SI, while participants learned to perform a visually cued wrist flexion/extension tracking task. Participants receiving real rTMS demonstrated greater errors in tracking during task acquisition and at a second testing session the following day when no rTMS was delivered [85]. Pleger et al (2006) used high-frequency rTMS over SI to investigate effects on SI and M1 physiology. Following 5 Hz rTMS, functional magnetic resonance imaging (fMRI) revealed that a cluster of voxels within ipsilateral M1 was shown to be negatively correlated with improvements in tactile frequency discrimination. Further, participants who showed the best perceptual performance had the greatest activation increases in SI and the lowest activation increases within M1. This study also demonstrated that 5 Hz rTMS over SI increases the effective connectivity between SI and M1 [89].

### 2.3. Repetitive TMS Considerations

One consideration in attempting to induce rTMS effects relates to monitoring muscle activity during stimulation. In one study, the effects of 5 Hz rTMS over M1 were modified by flexion or extension of the wrist during rTMS application [91]. SICI in flexor carpi radialis was decreased following rTMS paired with wrist flexion and increased with wrist extension. Similarly, SICI in extensor carpi radialis was decreased following rTMS paired with wrist extension and increased with wrist flexion [91]. No effects on ICF were observed. A recent review highlights evidence that neural activity prior to rTMS either by priming with an independent TMS protocol or voluntary muscle activation influences the after effects of the interventional rTMS protocol [92] suggesting metaplasticity. These data suggest that the state of muscle activity during rTMS can strongly modify select circuitry within M1.

Low-frequency rTMS over SI has been shown to have only slight, nonsignificant effects on SEPs recorded from ipsilateral SI [93]. Interestingly, median nerve stimulation followed by single-pulse TMS to M1 at ISIs of 150 ms has shown reductions in contralateral SI SEP components (N20p-P25 & P25-N33), possibly through secondary effects of corticocortical projections from the contralateral, non-stimulated M1 [94]. This suggests that SI changes may be more responsive to TMS manipulations of activity within M1 compared with those directly applied to SI.

Technical aspects of rTMS are an important consideration. In general, the coil orientation, stimulus intensity and frequency are factors that can be controlled by the experimenter. Tings et al. (2005) performed 5 Hz rTMS over M1 using both PA and AP orientations in separate sessions. Monophasic rTMS in the PA orientation induced facilitation of MEP amplitudes, whereas monophasic rTMS with AP orientation suppressed MEP amplitudes [95]. Berger et al. (2011) delivered 1 Hz rTMS at intensities of 40%, 80%, and 100% RMT. MEP amplitudes decreased for the lowest intensity, while no significant change was observed at 80% RMT, and facilitation was recorded at 100% RMT [96]. Quartarone et al. (2005) also showed an intensity-dependent relationship. No increase in MEP amplitude was found following rTMS at 90% AMT, but when intensity was increased to 90% RMT, MEP amplitude significantly increased over time [97]. Overall, the technical parameters of rTMS should be carefully considered in order to produce the desired after effect (for further review, see [98, 99]).

#### 2.3.1. Theta-Burst Stimulation

A novel form of repetitive TMS called theta-burst stimulation (TBS) is composed of bursts of three pulses delivered at 50 Hz and repeated at 5 Hz [100] and is designed to mimic LTP and LTD inducing paradigms in animal models [57, 101–104]. TBS can be applied in a continuous (cTBS) or an intermittent (iTBS) pattern to induce short-term plasticity changes within the cortex. CTBS involves uninterrupted bursts of TBS pulses over a short period of time, while iTBS consists of a 2-second train of TBS repeated every 10 seconds [100].

The neural mechanisms that mediate TBS effects in humans remain unclear, though information obtained from rat models is advancing our understanding. At the cellular level, TBS delivered over rat cortex alters the expression of glutamic acid isoforms GAD 65 and GAD 67 and GAT-1 [82]. GAT-1 is a presynaptic GABA transporter, GAD 65 is important for GABA synthesis for the purpose of neurotransmission [105], and GAD 67 supports GABA synthesis within the cytosol [106]. Two hours following TBS, GAD 65, and GAT-1 expression were increased, while GAD 67 was decreased [82]. One to seven days following stimulation opposite effects were found; GAD 65 and GAT-1 expression decreased while GAD 67 increased. The acute and longer-term effects were observed for both iTBS and cTBS [82]. These findings demonstrate that both TBS protocols promote GABA-related activity within targeted cortex. However, iTBS versus cTBS protocols appear to have different effects on the expression of cortical proteins involved in inhibitory cortical systems. Inhibitory neurons that influence the synchronization of pyramidal cells express parvalbumin (PV), while those that control dendritic input to pyramidal cells and other interneurons express calbindin D-28k (CB) [107]. ITBS reduced the number of PV-positive cells, while CTBS decreased the number of CB expressing cells [81]. The suggestion from these authors is that iTBS may target the inhibition of pyramidal cell output neurons, and cTBS alters the inhibitory activity of interneurons that control the synaptic inputs to pyramidal cells [81]. Collectively, the rat model has demonstrated that neural mechanisms of TBS involve changes to the inhibitory neuronal circuitry within the targeted cortex. It remains unclear whether the findings in the rat model are translatable to human TBS studies that typically employ a single-600-pulse TBS protocol.

In humans, evidence suggests that TBS paradigms may be related to LTP/LTD-like effects and GABAergic activity [108]. Using magnetic resonance spectroscopy (MRS), cTBS increased GABA concentration within targeted cortex [109], and it was suggested that cTBS at 80% AMT stimulates GABA_A_ interneurons whose elevated activity is maintained via GAD 65 expression, and later, by elevated GABA within the cytoplasm via GAD 67. In addition to altering cortical inhibition, evidence in humans also suggests that TBS modulates activity in glutamatergic systems. Although MRS revealed no change in glutamate concentration following cTBS [109], the effects of cTBS are abolished after NMDA receptor blockage [110, 111]. Therefore, cTBS protocols may alter activity in both inhibitory and excitatory circuitry. Dopamine also contributes to the mechanisms of iTBS and cTBS, and effects are abolished following D2 receptor blockage [112].

Several studies have examined TBS paradigms applied over M1. CTBS over M1 decreases MEPs for 20–60 minutes [100, 113, 114] and reduces SICI [100, 113, 115]. In contrast, iTBS applied over M1 increases MEP amplitude for 15–20 minutes [100, 114] and increases SICI [100, 115]. TBS provides an opportunity to modulate cortical excitability at both the site of stimulation and remote areas. For example, cTBS over M1 reduces MEP amplitude bilaterally [116]. In another study, cTBS over M1 decreases MEPs in the stimulated hemisphere and increases MEPs evoked from the nonstimulated M1 [115]. ITBS applied over M1 increases MEPs in the stimulated hemisphere [100, 115] and decreases MEP amplitude in the non-stimulated hemisphere [115].

Investigating the effects of TBS over SI, Ishikawa et al. (2007) delivered cTBS to left SI (2 cm posterior to M1) and recorded SEPs elicited from right and left median nerve stimulation. Following cTBS over left SI, a reduction in the amplitude of SEP components P22-N30 and P25-N33, elicited from the right but not left median nerve, was observed for 13 minutes following stimulation as measured by two time blocks at 0–3 and 10–13 minutes [116]. No significant suppression of SEP amplitude was observed at 20 minutes following stimulation [116]. To probe the perceptual effects of the reduced SEP amplitude following cTBS, Rai et al. 2011 (in press) examined tactile temporal and spatial amplitude discrimination thresholds before and following cTBS over left SI defined as a point 2 cm posterior to motor hotspot. In line with the reduction in SEP amplitude following cTBS over left SI [116], temporal discrimination threshold (TDT) and spatial amplitude discrimination threshold (SDT) were impaired, and thresholds were elevated following stimulation at certain time intervals for up to 18 minutes. It is notable that the psychophysical changes (up to 18 minutes) appear to slightly exceed the physiological changes (13 minutes) following cTBS. In particular, changes in TDT between 1–3 and 11–14 minutes are in line with SEP changes following cTBS [117]. A recent study explored SEPs and high-frequency oscillations (HFOs) before and following cTBS and found that HFOs only were suppressed at 15 minutes following stimulation [118].

Intermittent TBS applied over SI also provides the opportunity to modulate SI physiology and perception. Katayama and Rothwell (2007) applied iTBS over left SI (2 cm posterior to left M1) and measured SEPs elicited from the right median nerve. Following stimulation, SEP amplitudes (N200-N20p, N20p-P25, and P25-N33) were increased at 15 and 30 minutes following stimulation [119]. In another study, Premji et al., (2010) applied iTBS over left SI and measured SEPs elicited from the right and left median nerves before and at 5, 15, and 25 minutes following stimulation. The amplitude of the N20-P25 SEP was increased at 15 to 25 minutes in the stimulated SI and for 5 minutes in the non-stimulated hemisphere [120]. Similarly, iTBS applied to left SI facilitated SEPs at 15 and 30 minutes (N20o-N20p at 15 min; N20p-P25 at 15 and 30 minutes) [118]. In the same study, following iTBS, no changes were observed in early or late HFOs. Perceptual benefits have also been observed following iTBS over SI. An improvement in tactile spatial acuity on the right index finger was observed for up to 30 minutes following iTBS over left SI [121].

Continuous TBS has been used to investigate the influence of SI on the excitability within M1. Ishikawa et al., (2007) delivered cTBS over SI and observed that MEPs were unchanged. Our lab has recently furthered this investigation by probing the influence SI on corticospinal excitability, intracortical and interhemispheric motor circuitry for the representation of the first dorsal interosseous muscle (FDI) of the hand (paper in preparation). We observed that cTBS over left-hemisphere SI increases the corticospinal output of the contralateral hand (increased MEPs), leads to modest but insignificant increases in contralateral ICF and ipsilateral SIHI, and does not alter SICI. Importantly, the influence on corticospinal excitability is specific to the direction of induced current, a topic that will be discussed later within this paper, and relates to the discrepancy between previous findings [116] and ours.

In addition to the influence of SI, higher-order somatosensory loci may also influence M1 activity. One such area that has been a focus of interest in our lab is Brodmann's area 5. Using dual coil TMS, we observed that area 5 facilitates M1 output to the FDI muscle of the hand when the thumb and index finger receive tactile stimulation [122]. We recently investigated the influence of area 5 on M1 corticospinal excitability and circuitry [123]. CTBS, iTBS, and sham TBS were delivered over area 5, and MEPs, SICI, and ICF were measured from the FDI muscle on each hand for up to one hour following stimulation. MEPs were increased bilaterally following cTBS, increased in the contralateral hand following iTBS and unchanged in the sham group. ICF and SICI were unchanged [123]. Further, cTBS over left-hemisphere area 5 increases SIHI in the ipsilateral hand [124]. Our studies in area 5 have led to the conclusion that higher-order somatic loci provide powerful modulation over the corticospinal and transcallosal output of M1 neurons. Comparing the results of our studies from area 5 versus SI, it appears that higher-order loci have may have a more potent influence on M1 activity, at least by the measures we obtained.

### 2.4. TBS Considerations

Although cTBS and iTBS protocols have been in use since the original publication appeared in 2005 [100], evidence continues to accumulate that the after effects are not always as predicted. We observed that both iTBS, thought to induce LTP-like effects, and cTBS, thought to induce LTD effects, lead to the same outcome following delivery over area 5 [125]. Similarly, another study has shown that cTBS and iTBS delivered over SI act similarly, such that both cTBS and iTBS each act to reduce the amplitude of laser-evoked potentials [126].

The effects of TBS are also dependent on stimulus intensity and the direction of induced current within the cortex. Some studies have demonstrated a lack of excitability change, or an effect in the opposite direction to the original observations by Huang et al. (2005), and this may relate to the intensity of TBS delivered. In contrast to the MEP suppression following cTBS delivered at 80% active motor threshold [100], cTBS at 70% rest motor threshold increases MEP amplitude [127, 128]. Less intense cTBS and iTBS delivered at 70% active motor threshold did not alter MEP amplitudes [129]. Another important consideration is the direction of induced current in the cortex. TBS is a biphasic waveform paradigm that may be applied with a coil orientation to induce a posterior-to-anterior initial phase followed by anterior-to-posterior (PA-AP) current in the cortex or vice versa (AP-PA). When applied with an AP-PA-induced current flow, cTBS led to increased MEP amplitude in the ipsilateral hand, in contrast to PA-AP cTBS which had no bilateral effect [115]. CTBS delivered in the AP-PA orientation induced a stronger effect on MEP amplitudes when the two orientations were matched in absolute stimulus intensity [113]. Together, these studies indicate that the effects of TBS are determined by parameters of stimulus intensity and direction of induced current in the brain.

Metaplasticity is also a consideration for TBS protocols. Muscle activity preceding, during, or immediately following TBS may alter after effects. CTBS-induced suppression and iTBS-induced facilitation of MEPs were abolished when stimulation was applied during simultaneous 10% MVC of the target muscle [130]. In contrast, muscle contraction performed for 1 minute following iTBS enhanced MEP facilitation and reduced the MEP inhibition [130]. Similarly, facilitation of MEP amplitude was observed following 300-pulse cTBS [127]. However, facilitation was replaced with inhibition when cTBS was preceded by 5 minutes of isometric thumb contraction at ~25% MVC [127]. When a brief cTBS protocol of 150 pulses is delivered one minute after facilitatory iTBS, the enhancement effect is abolished [131]. Likewise, a brief iTBS protocol shortly following cTBS abolishes any depressive effects on MEP amplitude [131].

## 3. Additional TMS Protocols to Induce SI Plasticity

In monkeys, the excitability of M1 pyramidal tract neurons is altered in response to stimulation of peripheral nerves [132]. In humans, peripheral nerve stimulation also modulates corticospinal excitability and circuitry within M1. Corticospinal excitability is decreased for 20–1000 ms following median nerve stimulation [49], and with the addition of cutaneous stimulation to the digit, ICF is enhanced [133]. SICI is reduced following cutaneous stimulation of the index finger [134]. Magnetoencephalography studies have demonstrated that the 20 Hz rhythm generated in the motor cortex is increased 20–1000 ms (tested at intervals of 200 ms) following median nerve stimulation and suppressed during voluntary movement and motor imagery of the hand [135, 136]. At shorter intervals and in contrast to SAI and LAI, median nerve stimulation followed by single-pulse TMS to M1 at ISIs of 45–70 ms facilitates corticospinal excitability circuitry involved in ICF, while decreasing SICI, an effect named afferent-induced facilitation [137]. The aforementioned studies demonstrate that somatosensory afferent input is capable of transiently modifying M1 circuitry directed at muscles of the hand.

Longer-lasting changes in M1 excitability can be observed following manipulation of somatosensory afferent input. Blocking the peripheral afferent volley from synapsing within SI may also induce changes within M1. In humans, ischemic nerve block (INB) has been used to reproduce deafferentation, and TMS protocols may be used to examine changes in motor excitability. Compared to pre-INB measures, muscles proximal to the INB in the upper [138–140] and lower limb [140] demonstrate increased MEP amplitude, indicating that alterations in afferent input modulate the corticospinal excitability of M1. After INB, low-frequency rTMS (0.1 Hz) at a rate which does not induce excitability changes in the cortex in normal conditions significantly increased MEP amplitude, SICI, and ICF to a greater extent than INB alone [138]. However, in the same study, rTMS applied to the ipsilateral side of the INB canceled the MEP enhancement and decreased ICF, indicating M1 interhemispheric effects. The authors suggest that deafferentation permits the circuitry within M1 to be more susceptible to plastic changes [138]. Furthermore, MRS has clearly indicated that GABA, an inhibitory neurotransmitter, is decreased following INB in humans [141] leading the authors to suggest that INB-induced changes may involve a release of the SI to M1 inhibitory influence allowing the motor circuitry to be subsequently enhanced.

Another promising TMS-plasticity approach is called quadripulse stimulation (QPS) and involves four monophasic pulses delivered at 0.2 Hz for 30 minutes with effects that persist for 75 minutes following stimulation [142]. QPS is thought to modulate activity within M1 excitatory neural circuits [143, 144]. At short interpulse intervals ranging from 1.5 to 10 ms, MEPs are facilitated, while longer intervals (50, 100 ms) lead to a decrease in MEP amplitude. The greatest facilitation and suppression are observed at intervals of 5 and 50 ms, respectively [145]. One study has examined the use of QPS over M1 to alter excitability within contralateral SI as measured using SEPs [146]. The amplitude of the P25-N33 component was enhanced during both 5 and 50 ms QPS. However, at 30 minutes following QPS, only the 5 ms QPS paradigm led to a sustained increase in the P25-N33 that persisted for up to 90 minutes following stimulation [146]. These data suggest that QPS at 5 ms is a powerful modulator of SI excitability with effects that may outlast other TMS plasticity-inducing approaches. The threshold from LTD to LTP-like effects is modified by prior history of cortical activity [145], similar to the reversal of effects seen when voluntary contraction precedes TBS protocols [127].

## 4. Conclusion

This paper has illustrated the importance of understanding hand function through contributions of the somatosensory cortex using TMS plasticity-inducing protocols. Evidence clearly demonstrates that plasticity-inducing TMS protocols are a powerful tool to modulate SI physiology, tactile perception, and neural activity within M1. PAS, rTMS, and TBS are repetitive forms of TMS brain stimulation that may be used to alter the neurophysiology of cortical circuitry related to hand control. TMS paired with measures of physiology and/or perception further the understanding of the neural mechanisms that underpin somatosensory-guided hand control. This information is fundamental to creating new therapeutic applications of TMS plasticity protocols for clinical populations such as stroke and dystonia that present with impaired hand movement. An important consideration in all TMS techniques described in this paper is the state of neural activity within cortex prior to application of the plasticity-inducing stimulation since all protocols appear to be sensitive to metaplastic effects. Further, stimulus parameters such as intensity, orientation, and frequency influence the outcome of TMS protocols and are therefore important considerations in experimental design. Through further use of TMS plasticity-inducing protocols, we will continue to advance the understanding of sensorimotor hand control and further optimize protocols to evoke desired effects. This latter step will be a key element for future studies that aim to use plasticity-inducing TMS protocols as a potential therapeutic avenue to improve hand function.
